# ﻿Taxonomic delimitation and molecular identification of clusters within the species *Zanthoxylumnitidum* (Rutaceae) in China

**DOI:** 10.3897/phytokeys.196.79566

**Published:** 2022-05-12

**Authors:** Yunrui Qin, Renchuan Hu, Hui Zhao, Guiyuan Wei, Zhiqiang Lu, Yunfeng Huang

**Affiliations:** 1 Guangxi Institute of Chinese Medicine and Pharmaceutical Sciences, Nanning 530022, Guangxi, China Guangxi Institute of Chinese Medicine and Pharmaceutical Sciences Nanning China; 2 CAS Key Laboratory of Tropical Forest Ecology, Xishuangbanna Tropical Botanical Garden, Chinese Academy of Sciences, Mengla 666303, Yunnan, China South China Normal University Guangzhou China; 3 Guangdong Provincial Key Laboratory of Biotechnology for Plant Development, School of Life Sciences, South China Normal University, Guangzhou, 510631, China Xishuangbanna Tropical Botanical Garden, Chinese Academy of Sciences Mengla China; 4 Guangxi Key Laboratory of Traditional Chinese Medicine Quality Standards, Nanning 530022, Guangxi, China Guangxi Key Laboratory of Traditional Chinese Medicine Quality Standards Nanning China

**Keywords:** Classification and identification, Liang-Mian-Zhen, medicinal materials, taxonomic delimitation

## Abstract

*Zanthoxylumnitidum*, known as Liang-Mian-Zhen in China, is a traditional Chinese medicinal plant used to treat traumatic injury, rheumatism, paralysis, toothache, stomach ache, and venomous snake bites. Two varieties of the species have been described and three morphological types have been reported within the original variety. However, taxonomic delimitation and molecular markers for distinguishing these varieties and types within this species remain unknown. Since different populations exhibit varying chemical compositions, easy identification of intraspecific taxa is crucial. We collected 420 individuals from 38 natural populations, 3 samples of standard medicinal material, and 17 folk-medicine samples to perform classification and identification within *Zanthoxylumnitidum*. Four distinct genetic clusters (A, B, C, and D) were highly supported by the nuclear barcode. Two distinct chloroplast clusters (A1 and A2) were further detected within A, and three others had one-to-one correspondence with the remaining nuclear clusters. Molecular identification showed that the 17 folk samples comprised A1, A2, B, and D, while the 3 standard samples belonged to A2. The internal transcribed spacer (ITS) region and *rbc*L gene are proposed as barcodes for rapid and accurate identification of the different Liang-Mian-Zhen lineages in China. This study highlights the importance of accurate taxonomic delimitation in combination with rapid and accurate molecular identification of medicinal plants.

## ﻿Introduction

Medicinal plants are widely used in clinical medications, daily chemical products, and food and drink products in China, and play an important role in people’s daily life (Zhu et al. 1983; Hawkins 2008; Bansal et al. 2018; Lu et al. 2020). With the increase in human population and the decrease in natural medicinal resources in recent decades, the market demand for cultivated medicinal plants has been expanding (Zhou and Wu 2006; Hawkins 2008; Techen et al. 2014). Moreover, many substitutes are being used, although their active ingredients are distinctly different from conventionally used substances (Sui et al. 2011; Chao et al. 2014; Dubey and Sawant 2015; Qin et al. 2019). Generally, the active ingredients of any substance are considered to be related to different genetic (or morphological) clusters or species (e.g., Cirak et al. 2007, 2013; Chao et al. 2014; Fan et al. 2015). In our previous study, such a phenomenon was observed among strains within the medical plant *Zanthoxylumnitidum* (Qin et al. 2019). Nine populations covering four morphological clusters were compared and significant differences among the chemical ingredients (e.g., nitidine chloride) were discovered. This suggests that not all *Z.nitidum* proveniences are equally suitable for medicinal use and the identification of intraspecific taxa is crucial.

*Zanthoxylumnitidum*, known as Liang-Mian-Zhen (两面针) in China, differs from congeneric species in terms of the characteristics of its perianth in two series, 4-merous flowers, 4-carpelled gynoecia, axillary and terminal inflorescences (thyrsiform) pedicel length (rarely reaching 1 cm in fruits), fruit follicles (to 0.9 cm and containing neither prickles nor trichomes), outer part of pericarp (not wider than endocarp), leaflet blades (without oil glands along secondary veins), and leaflet apex retuse (at the tip). Besides the typical variety (Z.nitidumvar.nitidum), a second variety, Z.nitidumvar.tomentosum, has been accepted (Hartley 1966; Editorial Committee of Flora 1997; Zhang et al. 2008; Chinese Pharmacopoeia Commission 2020). Three morphological types have also been proposed for Z.nitidumvar.nitidum (Editorial Committee of Flora 1997). A recent study reported that morphological boundaries between varieties or types within this species should be re-evaluated based on prickle appearance and the density of prickles on the trunk (Qin et al. 2019). In total, the morphological taxonomy of Chinese Liang-Mian-Zhen is disordered owing to the abundance of phenotypic variations and the use of different taxonomic methods (Editorial Committee of Flora 1997; Zhang et al. 2008; Qin et al. 2019). Hence, taxonomic delimitation within the medicinal plant Liang-Mian-Zhen in China remains controversial and more work is needed to disentangle its intraspecific structure.

Molecular identification is widely used in the authentication of medicinal plants (representing a morphological or genetic cluster) [Guo et al. 2013; Chao et al. 2014; Chen et al. 2015; Osathanunkul et al. 2016; Xin et al. 2018]. Short sequences from a standardized position of the genome are aligned and compared with a known DNA barcode reference library to identify the taxa to which a sample belongs (Chen et al. 2015; Liu et al. 2018). In contrast to traditional taxonomy, there are many advantages to molecular identification using DNA barcodes, such as objectivity, speed, and accuracy, making it more suitable for forensic applications (Chao et al. 2014; Chen et al. 2015). Ideally, an unknown sample can be identified as a specific species – usually as a genetic cluster or lineage – using its DNA barcode sequences based on a reliable reference library (Liu et al. 2018). In practice, before DNA barcode reference libraries are constructed, initial identification based on morphological traits is presupposed to be accurate (Chen et al. 2015). Thus, if taxonomic boundaries of closely related taxa are unresolved, inaccurate reference libraries will be produced, thereby impairing unbiased identification (Liu et al. 2018). Many case studies have reflected this phenomenon in which the taxonomic results based on morphological and molecular evidence are highly inconsistent with traditional taxonomic results (Hu et al. 2015; Su et al. 2015; Lu et al. 2021). Thus, genetic classification using short sequences with high discrimination power, along with the use of barcodes as critical markers for accurate identification, can greatly improve the accuracy of further molecular identification.

Liang-Mian-Zhen is traditionally used in Chinese medicine to treat traumatic injury, rheumatism, paralysis, toothache, stomach ache, and venomous snake bites (Yang and Chen 2008; Feng et al. 2011; Chinese Pharmacopoeia Commission 2020). Many active ingredients are extracted from this species, including nitidine chloride, chelerythrine, ethoxychelerythrine sanguinarium chloride, allocryptopine, and neoherculin (Qin et al. 2019). Owing to its antiviral, anti-inflammatory, analgesic, and antifungal effects, Liang-Mian-Zhen is widely used in the production of daily chemical products (e.g., toothpaste) and clinical medication (e.g., Sanjiuweitai capsules and Liang-Mian-Zhen Zhen Tong tablets); as such, a large number of Liang-Mian-Zhen materials are consumed every year in China (Yang and Chen 2008; Feng et al. 2011; Lu et al. 2020; Qin et al. 2020). However, Liang-Mian-Zhen classification is still controversial in China. Previous studies have shown that DNA barcodes based on nuclear and chloroplast barcodes have large differentiation between strains within *Z.nitidum* (Chen et al. 2013; Ma et al. 2014). Hence, DNA barcodes should be considered as molecular markers to delimit boundaries of different strains within Liang-Mian-Zhen.

In the present study, we conducted extensive sampling of the natural populations of Liang-Mian-Zhen in China, collected standard samples from China Resources Sanjiu, a large state-owned pharmaceutical company, and visited indigenous communities in different geographical regions to obtain folk medicinal samples. Our aim was to establish a standard for the resource classification and identification of Chinese Liang-Mian-Zhen, which will provide a guide for quality, safety, and efficacy during the utilization of medicinal materials. To this end, we asked the following questions: (1) How many genetic clusters can be delimited within this species in China based on nuclear and chloroplast barcodes? (2) Which is the most suitable taxonomic scenario among those currently available? (3) Which barcodes have high discrimination power and are suitable for intraspecific classification and identification of Liang-Mian-Zhen clusters? (4) Which clusters do the Liang-Mian-Zhen used by indigenous people belong to?

## ﻿Material and methods

### ﻿Sample collection and specimen identification

**Figure 1.**
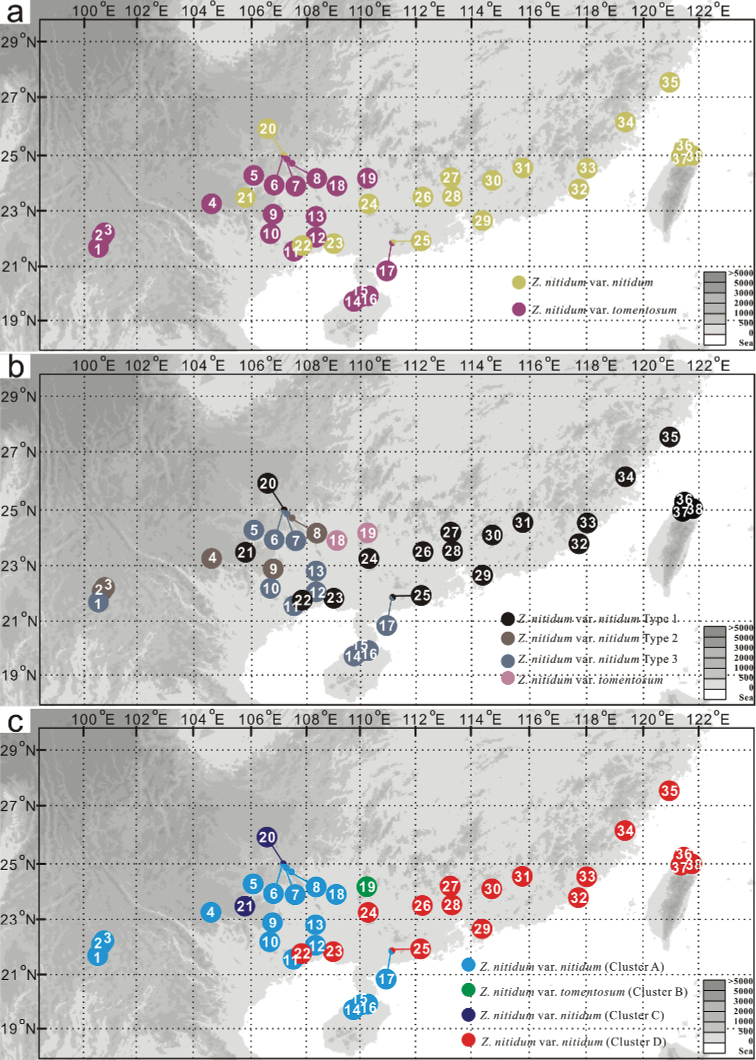
Geographical distribution of 38 *Z.nitidum* populations in China and its taxonomic scenarios currently used based on the presence of hairy or nearly glabrous leaf and rachis in Zhang et al. (2008) (**a**), the description of the Editorial Committee of Flora (1997) (**b**), and trunk appearance in Qin et al. (2020) (**c**).

**Figure 2.**
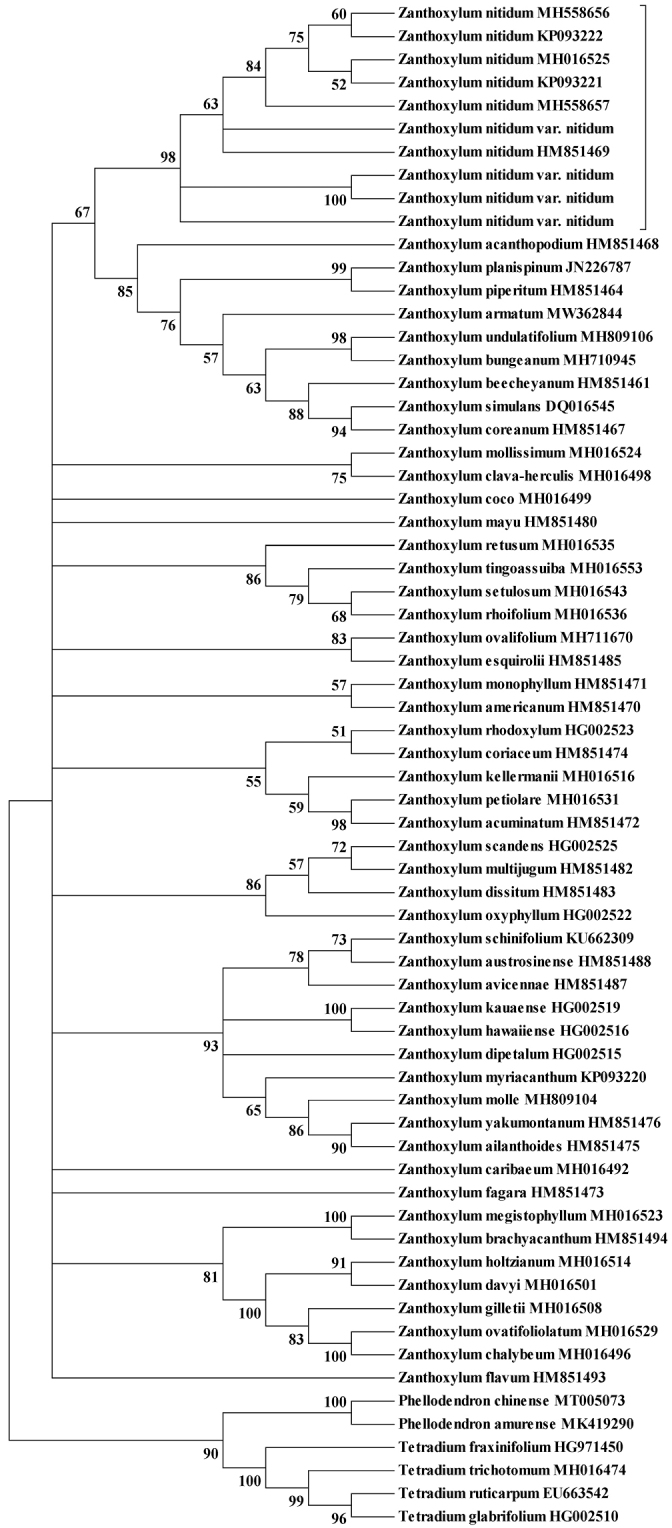
Maximum likelihood (ML) tree of *Zanthoxylum* species based on nuclear internal transcribed spacer (ITS) sequences.

We consulted the specimen records of *Z.nitidum* (including two varieties) available in the Chinese Virtual Herbarium (CVH, http://www.cvh.ac.cn/) and also examined all specimens of this species deposited in GXMI of Guangxi Institute of Chinese Medicine and Pharmaceutical Sciences and IBSC of South China Botanical Garden, CAS. Then, we conducted extensive sampling across its whole distribution range in China according to the consulted specimen records to collect enough samples for genetic analyses. All sampled individuals of the same population were spaced more than 50 m apart. Fresh leaves were collected for DNA sequencing and immediately placed in plastic sealed bags with sufficient silica gel to avoid DNA degradation. We also took photos of the trunks, leaves, branchlets and inflorescence of each individual for initial identification. We identified samples of this monophyletic species based on three taxonomic criteria (Figs 1, 2). All voucher specimens and dried leaves for DNA extraction were deposited in GXMI. Geographical information for each location was recorded using a HOLUX M-241 GPS Track instrument (Technology, Inc., Taiwan, China). Finally, 420 individuals from 38 natural populations across the entire distribution in China were collected for genetic analyses (Table 1).

**Table 1.** Sampling and classification information of *Zanthoxylumnitidum* from natural populations.

Pop. code	Location	GPS Coordinates	Altitude (m)	No. of individuals in internal transcribed spacer (ITS) cluster/type	No. of individuals in cpDNA lineage/haplotype
01 (ML)	Menglun, Yunnan	21.6911, 100.6472	558	2 (A-type5)	2 (A1-H1)
02 (MY)	Mengyang,Yunnan	22.1031, 100.7511	753	5 (A-type5)	5 (A2-H2: A2-H3 = 3:2)
03 (DDG)	Dadugang,Yunnan	22.2019, 100.8806	761	5 (A-type5)	12 (A2-H2: A2-H3 = 1:11)
04 (XC)	Malipo, Yunnan	23.2050, 104.9419	532	2 (A-type6)	5 (A2-H4)
05 (TL)	Tianling,Guangxi	24.2600, 106.0457	479	4 (A-type1)	4 (A2-H2)
06 (TEW)	Tiane, Guangxi	25.0175, 107.1616	535	6 (A-type1)	8 (A2-H2)
07 (TEL)	Tiane, Guangxi	24.9829, 107.1962	260	8 (A-type8:A-type11:A-type12 = 2:3:3)	18 (A2-H2)
08 (ND)	Nandan, Guangxi	24.8437, 107.3397	264	1 (A-type1)	2 (A2-H2)
09 (XL)	Daxin, Guangxi	22.9145, 106.7631	269	6 (A-type1)	11 (A2-H3)
10 (LZ)	Longzhou, Guangxi	22.3742, 106.6117	241	3 (A-type1:A-type6 = 2:1)	5 (A2-H3)
11 (DZ)	Fangcheng, Guangxi	21.6202, 107.5226	261	4 (A-type2:A-type5 = 2:2)	9 (A2-H2)
12 (GT)	Guitai, Guangxi	22.0799, 108.2121	101	9 (A-type3)	13 (A2-H2)
13 (NN)	Nanning, Guangxi	22.9070, 108.2160	160	9 (A-type1)	16 (A2-H2)
14 (HDZ)	Danzhou, Hainan	19.6104, 109.7366	120	8 (A-type2)	14 (A1-H1)
15 (LG)	Chengmai, Hainan	19.8144, 110.1064	47	12 (A-type7:A-type9:A-type10 = 6:3:3)	18 (A1-H1)
16 (HK)	Haikou, Hainan	19.9241, 110.2092	120	12 (A-type4)	19 (A1-H1)
17 (MM)	Maoming, Guangdong	21.8909, 111.1283	84	10 (A-type4:A-type6:A-type8 = 6:2:2)	10(A1-H1:A2-H2 = 6:4)
18 (BY)	Laibing, Guangxi	23.9677, 109.1837	290	9 (A-type2:A-type3 = 6:3)	20 (A2-H2)
19 (JX)	Jinxiu, Guangxi	24.1147, 110.1915	914	20 (B-type1:B-type2:B-type3:B-type4 = 7:6:4:3)	28 (B-H5)
20 (TE)	Tiane, Guangxi	25.0175, 107.1616	535	15 (C-type1:C-type4:C-type6:C-type7 = 9:3:2:1)	28 (C-H6:C-H7 = 20:8)
21 (NP)	Napo, Guangxi	23.3850, 105.8555	1026	13 (C-type2:C-type3:C-type5 = 7:4:2)	15 (C-H6)
22 (FL)	Fulong, Guangxi	21.8489, 107.8954	369	3 (D-type1)	3 (D-H8)
23 (NW)	Qinzhou, Guangxi	21.8856, 108.9255	343	10 (D-type2:D-type5 = 6:4)	13 (D-H8)
24 (BS)	Guiping, Guangxi	23.2028, 110.2032	478	10 (D-type1:D-type2:D-type6 = 3:4:3)	14 (D-H8)
25 (MM)	Maoming, Guangdong	21.8909, 111.1283	84	3 (D-type2)	3 (D-H8)
26 (ZQ)	Zaoqin, Guangdong	23.4534, 112.2185	384	5 (D-type5:D-type7 = 3:2)	7 (D-H8)
27 (YD)	Yingde, Guangdong	24.2411, 113.2146	52	6 (D-type1:D-type2 = 4:2)	10 (D-H8)
28 (GZ)	Guangzhou, Guangdong	23.5139, 113.2190	80	2 (D-type6)	2 (D-H8)
29 (SZ)	Shengzhen, Guangdong	22.6479, 114.3187	275	6 (D-type2:D-type6 D-type7 = 3:1:2)	11 (D-H8)
30 (HY)	Heyuan, Guangdong	24.1123, 114.7886	136	9 (D-type6:D-type8 = 5:4)	18 (D-H8)
31 (MZ)	Meizhou, Guangdong	24.5358, 115.8660	159	7 (D-type1:D-type2:D-type5 = 3:2:2)	12 (D-H8)
32 (ZZ)	Zhangzhou, Fujian	23.7805, 117.6242	10	12 (D-type1:D-type5 = 9:3)	16 (D-H8)
33 (XM)	Xiamen, Fujian	24.4993, 118.0951	202	8 (D-type1:D-type2 = 5:3)	15 (D-H8)
34 (FZ)	Fuzhou, Fujian	26.1026, 119.3183	65	8 (D-type3:D-type4 = 7:1)	9 (D-H8)
35 (RA)	Ruian, Zhejiang	27.4641, 121.0822	44	10 (D-type4)	18 (D-H8)
36 (DSK)	Danshuikou, Taiwan	25.1811, 121.4717	5	1 (D-type3)	1 (D-H8)
37 (WH)	Wanhua, Taiwan	25.0317, 121.5094	20	4 (D-type3)	4 (D-H8)
38 (NG)	Nangang, Taiwan	25.0439, 121.6097	60	2 (D-type4)	2 (D-H8)

### ﻿DNA extraction, amplification, and sequencing

The genomic DNA of all samples was extracted from approximately 15 mg of silica gel-dried leaves using the CTAB method (Doyle and Doyle 1990). The quality and concentration of genomic DNA were measured using a NanoDrop 1000 spectrophotometer. Qualified DNA was defined as having values of both 260/280 and 260/230 located in the range 1.6–2.1. Then, each DNA solution was diluted or concentrated to approximately 50 ng/µL for PCR amplification.

Owing to its high discrimination power, nuclear internal transcribed spacer (ITS) sequence fragments are frequently used for plant classification (CBOL Plant Working Group 2011; Pang et al. 2011; Lu et al. 2016), and have been proposed as core barcodes (CBOL Plant Working Group 2011). As core chloroplast barcodes (CBOL Plant Working Group 2009), *mat*K and *rbc*L are widely used for most seed plants (Levin et al. 2003; Kress and Erickson 2007; Fazekas et al. 2008; Kress et al. 2009; Su et al. 2015; Amandita et al. 2019). Other chloroplast fragments (e.g., *ycf*6-*trn*C and *pet*A-*psb*J) have also been used for phylogenetic studies (Shaw et al. 2007; Liu et al. 2012; Sokoloff and Gillespie 2012). Therefore, we used universal primers for the nuclear ITS fragments and four chloroplast DNA fragments (*mat*K, *rbc*L, *ycf*6-*trn*C, and *pet*A-*psb*J) to amplify and sequence each of the qualified DNA samples (Table 2). The primers were selected based on information from previous studies (White et al. 1990; Kress and Erickson 2007; Shaw et al. 2007; Kress et al. 2009; Liu et al. 2012; Amandita et al. 2019). PCR amplification was performed in a 50 µL reaction system with 2 µL of the prepared DNA, 5 µL of 10 × PCR buffer, 0.8 µL of dNTPs (2.5 mmol/ml), 4 µL of the primer, 0.4 µL of rTaq polymerase, and 41.5 µL of ddH_2_O. The reaction was programed according to the following procedure: initial template denaturation at 94 °C for 4 min, followed by 38 cycles of 94 °C for 40 s, 48 °C (*pet*A-*psb*J), 52 °C (*mat*K), 55 °C (*rbc*L), 59 °C (*ycf*6-*trn*C) or 60 °C (ITS) for 45 s, and 72 °C for 1.5 min, and final extension at 72 °C for 10 min.

**Table 2.** Primers used in this study.

Primer	Primer sequence	Tm (˚C)	Reference
ITS4	TCCTCCGCTTATTGATATGC	52	White et al. 1990
ITS5	GGAAGTAAAAGTCGTAACAAGG		White et al. 1990
*mat*K3F	CGTACAGTACTTTTGTGTTTACGAG	52	Amandita et al. 2019
*mat*K1R	ACCCAGTCCATCTGGAAATCTTGGTTC		Amandita et al. 2019
*rbc*LF	ATGTCACCACAAACAGAGACTAAAGC	55	Kress and Erickson 2007
*rbc*LR	GTAAAATCAAGTCCACCRCG		Kress et al. 2009
*psb*JF	ATAGGTACTGTARCYGGTATT	48	Shaw et al. 2007
*pet*AR	AACARTTYGARAAGGTTCAATT		Shaw et al. 2007
*trn*CF	CCAGTTCRAATCYGGGTG	59	Liu et al. 2012
*ycf*6R	GCCCAAGCRAGACTTACTATATCCAT		Liu et al. 2012

All PCR products were examined by agarose gel electrophoresis and photographed using a UV transilluminator. The products were purified using a TIANquick Midi Purification Kit (TIANGEN, Beijing, China) and the reaction mixtures were sequenced on an ABI 3130xl automated sequencer (Applied Biosystems, Foster City, California, USA). For DNA products that failed direct sequencing, we repeated the extraction, amplification, or sequencing experiment at least three times.

### ﻿Genetic classification of natural Liang-Mian-Zhen resources in China

We aligned and manually corrected nuclear ITS sequences and chloroplast sequences using MEGA 5 (Tamura et al. 2011). We first analyzed the haplotype relationship using NETWORK 10.0, based on the Median-Join method, for single-barcode and four-chloroplast fragments combined (Bandelt et al. 1999). Owing to the presence of some heterozygous sites in the nuclear ITS dataset, we phased these sequences using DnaSP v5 with default parameters (Librado and Rozas 2009). In addition, we constructed an ML tree of the *Zanthoxylum* species based on ITS sequences downloaded from NCBI (https://www.ncbi.nlm.nih.gov/), using MEGA 5 (Tamura et al. 2011) to further demonstrate the distinctiveness of *Z.nitidum* to the congeneric species. We further identified operational taxonomic units (OTUs) based on DNA barcodes using the Automatic Barcode Gap Discovery (ABGD) method (Puillandre et al. 2012). Indels or heterozygous sites of each fragment were deleted. The ABGD analysis was conducted based on information available online (https://bioinfo.mnhn.fr/abi/public/asap/asapweb.html). We used the Kimura (K80) ts/tv model and set X = 1.5 as the relative barcode gap width. The remaining parameters were retained as the default parameters.

### ﻿Molecular identification of Liang-Mian-Zhen samples

After genetic classification, we used the barcodes of each nuclear cluster or chloroplast cluster as references for subsequent molecular identification. The Liang-Mian-Zhen used by China Resources Sanjiu was treated as the standard reference because it has a rapid growth rate, a high level of nitidine chloride (Han et al. 2013; Qin et al. 2019), and is widely cultivated. We then visited 10 indigenous individuals who sold Liang-Mian-Zhen in different folk markets in Hainan, Guangxi, and Guangdong, with 1–4 samples obtained from each. We collected only a small number of broken leaves and voucher specimens without the trunk from both China Resources Sanjiu and the indigenous individuals, thereby making visual identification difficult. In total, 20 Liang-Mian-Zhen samples, including 3 standard samples and 17 samples of folk medicinal materials, were used for molecular classification and identification (Table 3). Total DNA was extracted, amplified, and sequenced according to the methods described above. For rapid identification, we directly used the aligned consistent sequences to construct ML trees using MEGA 5 (Tamura et al. 2011). The closely related species *Zanthoxylumarmatum* was chosen as an outgroup for phylogenetic analyses within *Z.nitidum* (Accession numbers: MW362848 and MN080708; Chen et al. 2013; Appelhans et al. 2018). Finally, we classified and identified the samples using the nuclear ITS barcode and four chloroplast barcodes.

**Table 3.** Sampling information and molecular identification results of folk and standard Liang-Mian-Zhen samples.

Code	Location	Internal transcribed spacer (ITS) cluster and No. of individuals	cpDNA lineage/haplotype and No. of individuals
Folk-DX	Dongxing, Guangxi	2 (A:D = 1:1)	2 (A2-H2:D-H8 = 1:1)
Folk-JX	Jingxi, Guangxi	1 (A)	1 (A2-H3)
Folk-NM	Ningming, Guangxi	1 (A)	1 (A2-H3)
Folk-PX-1	Pingxiang, Guangxi	1 (A)	1 (A2-H3)
Folk-PX-2	Pingxiang, Guangxi	1 (A)	1 (A2-H3)
Folk-LZ	Longzhou, Guangxi	1 (A)	1 (A2-H3
Folk-DY	Dayaoshan, Guangxi	2 (B)	2 (B-H5)
Folk-MM-1	Maoming, Guangdong	4 (A:D = 3:1)	4 (A1-H1:D-H8 = 3:1)
Folk-MM-2	Maoming, Guangdong	2 (D)	2 (D-H8)
Folk-HK-1	Haikou, Hainan	2 (A)	2 (A1-H1)
CRS-standard	China resources Sanjiu	3 (A)	3 (A2-H3)

## ﻿Results

### ﻿Genetic classification

**Figure 3.**
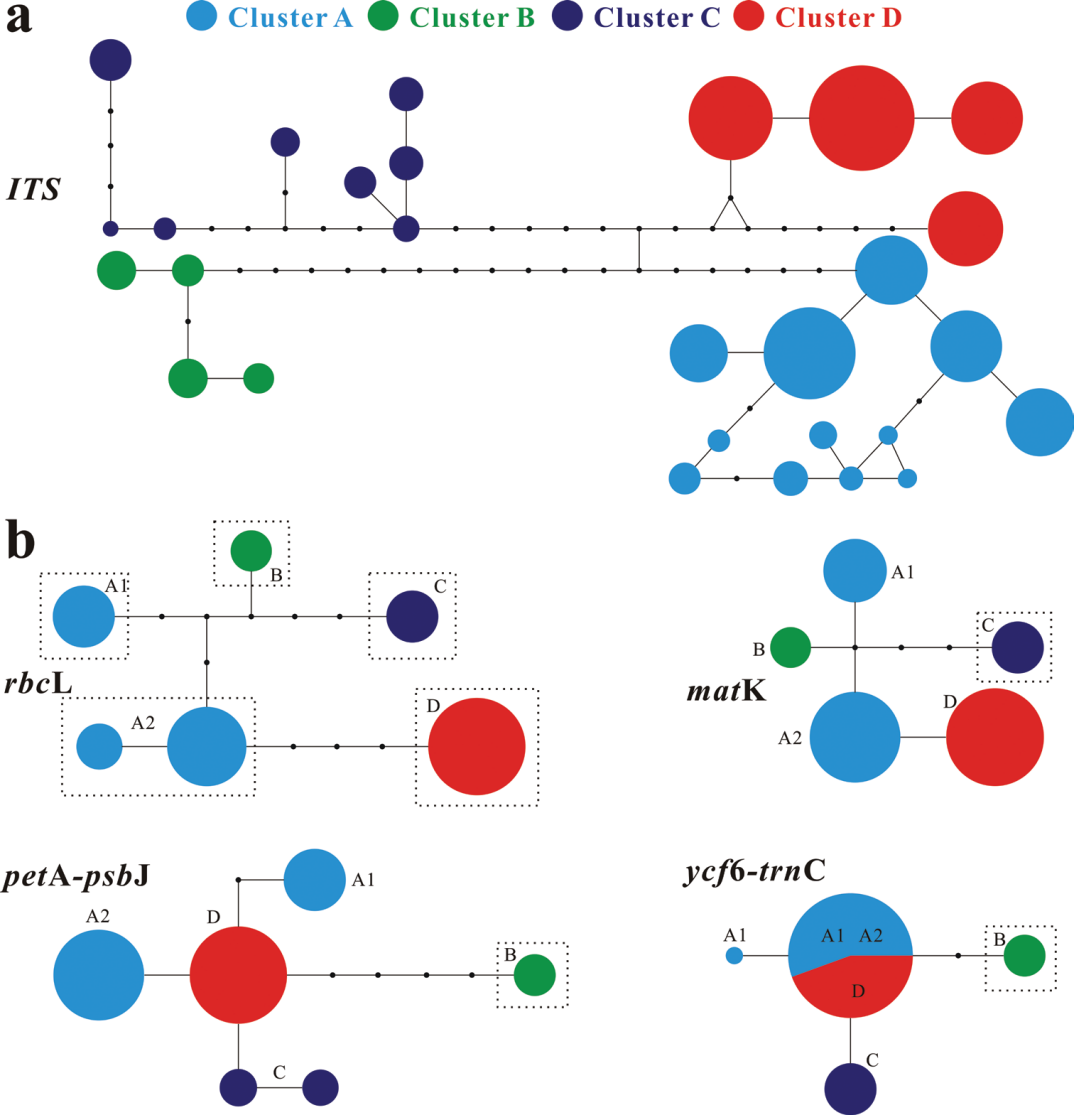
Network analysis of each barcode. Circle size is proportional to the haplotype frequency. Different colors indicate different clusters. The four clusters correspond to four morphological clusters in Fig. 1c.

We obtained 269 ITS sequences from 420 individuals across 38 natural populations of *Z.nitidum* in China (Fig. 1, Table 1). We recovered 31 different ITS types, and the aligned sequences were 658 bp in length (Table 1). Four genetic clusters (A, B, C, and D) were detected based on the network analysis of these ITS sequences (Fig. 3a). Cluster D was widely distributed across Guangxi, Guangdong, Zhejiang, Fujian, and Taiwan. Cluster C was distributed in North and Southwest Guangxi. Cluster B was narrowly distributed in northeastern Guangxi. Cluster A was widely distributed across Yunnan, Guangxi, Hainan, and Guangdong, and its distribution partially overlapped with that of clusters C and D (Figs 1c, 3a). These genetic classification results are consistent with the third taxonomic scenario based on trunk appearance (Fig. 1c). Phylogenetic analysis confirmed that Liang-Mian-Zhen formed a clade distinct to congeners as the downloaded ITS sequences covered approximately a quarter of the *Zanthoxylum* species (Fig. 2).

**Figure 4.**
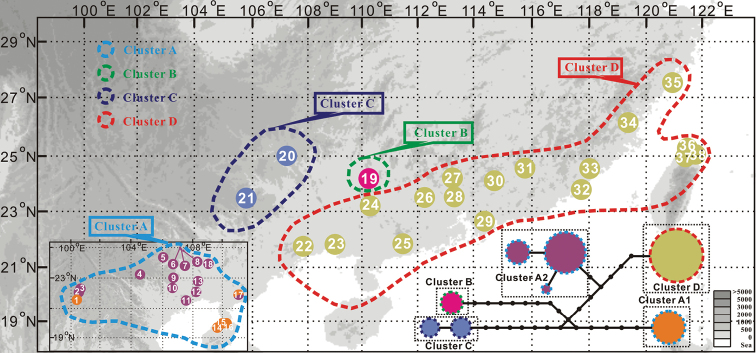
Distribution and network of five chloroplast lineages recovered from *Zanthoxylumnitidum* in China. Dotted lines of different colors indicate different nuclear clusters. Circle size is proportional to the haplotype frequency. Different colored circles indicate different chloroplast clusters.

After sequencing all four chloroplast barcodes (*mat*K, *rbc*L, *ycf*6-*trn*C, and *pet*A-*psb*J), the aligned sequences of these barcodes were 703, 533, 600, and 1040 bp in length, respectively. In total, 33 substitutions were detected across the 4 chloroplast barcodes. Only 8 cpDNA haplotypes were recovered from the 420 individuals across 38 natural populations, and these haplotypes were divided into 5 clusters (A1, A2, B, C, and D; Fig. 4, Table 1). All five chloroplast clusters were distributed across different geographical regions. Chloroplast clusters B, C, and D corresponded to the three nuclear clusters B, C, and D, respectively. However, two different chloroplast clusters (A1 and A2) were found within A. A1 was mainly found in Hainan and partially extended to southern Guangdong. In addition, it was also found in a preserved individual (coded as pop 1 in table 1) in the Xishuangbanna Tropical Botanical Garden (XTBG; Menglun, Yunnan). A2 was found in the remaining populations within A. We did not find distinct morphological differences between A1 and A2 from voucher specimens based on leaf shape and size, trunk appearance, and flowers. Accession numbers of all nuclear and chloroplast sequences are from MZ769312 to MZ769362 and from MZ773282 to MZ773393, respectively.

**Figure 5.**
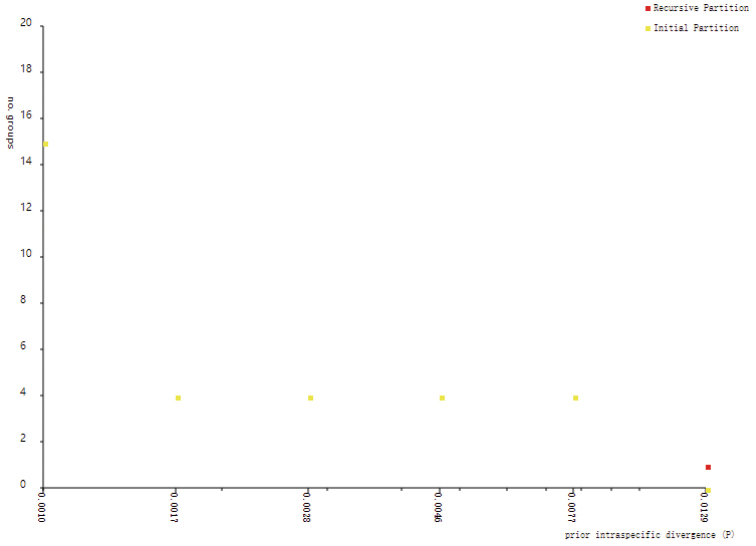
Genetic classification based on nuclear internal transcribed spacer (ITS) sequences using the Automatic Barcode Gap Discovery (ABGD) method.

**Figure 6.**
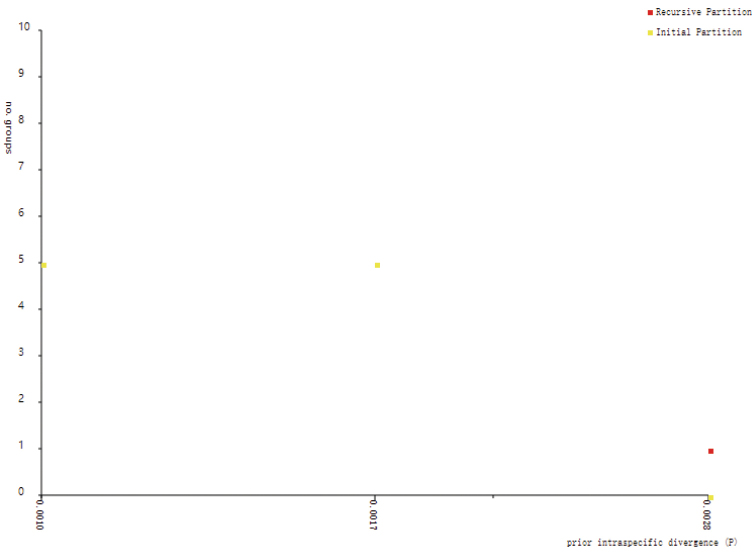
Genetic classification based on four chloroplast barcodes combined using the Automatic Barcode Gap Discovery (ABGD) method.

Additionally, the four OTUs recognized by the ABGD analysis were consistent with four clusters based on the network analysis (Figs 3a, 5), the results of which were highly inconsistent with the two past taxonomic scenarios (Fig. 1a, b) but highly consistent with the recent taxonomic scenario based on trunk appearance (Fig. 1c). For the combined four-chloroplast fragments, the five unveiled chloroplast clusters based on the network analysis were also revealed by the ABGD method (Figs 4, 6). The single chloroplast barcode, *rbc*L, produced the same result as the four fragments combined (Figs 3b, 4). The *mat*K gene only distinguished C from a group comprising the remaining nuclear or chloroplast clusters. Additionally, the other two chloroplast barcodes (*ycf*6-*trn*C and *pet*A-*psb*J) similarly classified this species into two groups: one represented by B, and the other comprising the remaining clusters.

### ﻿Molecular identification

**Figure 7.**
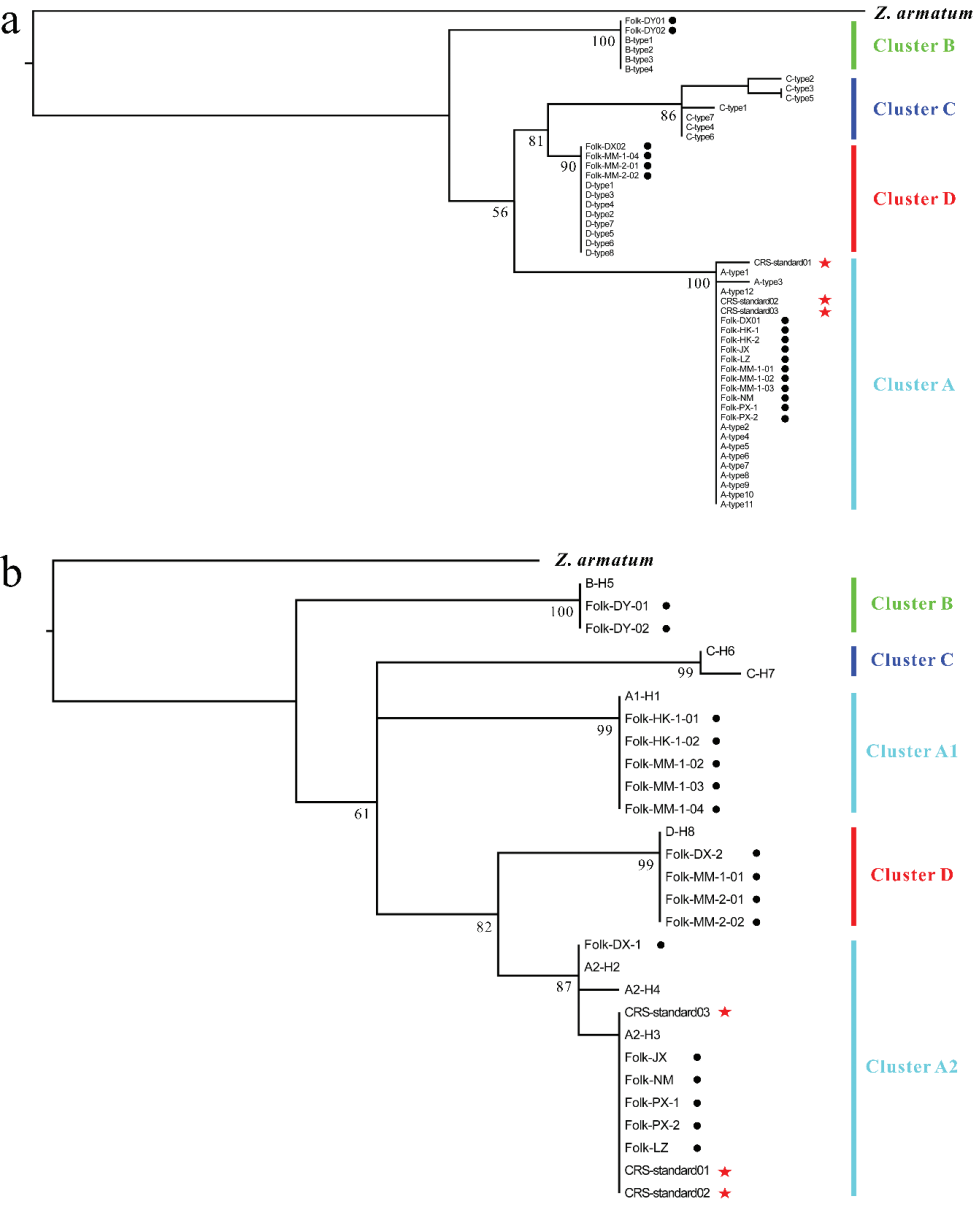
Molecular identification of the Liang-Mian-Zhen samples using Maximum likelihood (ML) trees based on nuclear internal transcribed spacer (ITS) barcodes (**a**) and four chloroplast fragments combined (**b**). Red stars indicate the standard samples obtained from China Resources Sanjiu and black dots indicate folk medicinal samples collected from indigenous individuals in different geographical regions.

Molecular identification was determined via phylogenetic analyses. During identification, each sample was delimited into the closest cluster. Four nuclear clusters (A, B, C, and D) and five chloroplast clusters (A1, A2, B, C, and D) were strongly supported by phylogenetic analyses (Fig. 7). The three standard Liang-Mian-Zhen samples from China Resources Sanjiu were identified as cluster A based on the nuclear ITS barcode and as A2 based on the four chloroplast fragments. However, for the 17 folk medicinal samples, three clusters (A, B, and D) were identified based on the nuclear ITS barcode. Four chloroplast clusters (A1, A2, B, and D) were identified based on the four chloroplast fragments. Interestingly, the molecular identification results for the folk medicinal samples were related to the natural distribution of *Z.nitidum* based on nuclear and chloroplast barcodes (Figs 1, 7, Table 1). For example, two folk Liang-Mian-Zhen samples from Jinxiu (Guangxi) were identified as cluster B based on their nuclear ITS sequences and cluster B based on four chloroplast fragments. Similarly, folk Liang-Mian-Zhen samples collected from Guangdong were assigned to nuclear clusters A or D and chloroplast clusters A2 or D. Folk samples collected from southern Guangxi were identified as A or D and A2 or D, and folk samples from Hainan were identified as clusters A and A1. In addition, all folk Liang-Mian-Zhen samples shared chloroplast haplotypes with nearby natural populations (Table 1 and Table 3).

## ﻿Discussion

### ﻿Three well-delimited clusters

In this study, we examined the intraspecific delimitation of the commercial medicinal plant *Z.nitidum* using 5 DNA barcodes based on samples from 38 natural populations (Fig. 1). The nuclear ITS sequences classified the species into four distinct genetic clusters (A, B, C, and D; Figs 3a, 5). This finding is consistent with a recent taxonomic scenario based on trunk appearance (Editorial Committee of Flora 1997; Zhang et al. 2008; Qin et al. 2019). However, chloroplast delimitation based on 4 fragments classified the 38 natural populations of this species into 5 clusters (A1, A2, B, C, and D; Figs 4, 6). The chloroplast clusters B, C, and D correspond one-to-one to the three nuclear clusters B, C, and D. In addition, they also correspond to three different geographical groups. Previous empirical studies have suggested that morphological taxonomy can be supported by molecular evidence (Su et al. 2015; Lu et al. 2021). According to the integrative taxonomic method (Su et al. 2015; Lu et al. 2021; Parker et al. 2021), taxonomic boundaries for clusters B, C, and D were demarcated based on the above evidence and form three well-delimited clusters. However, cluster A identified by nuclear barcode is split into two distinct chloroplast clusters, A1 and A2 (Fig. 4).

Chloroplast cluster A2 is phylogenetically and geographically closer to D, which is a possible indicator for introgression from D to A (Arnold 1997; 2006; Abbott 2017; Lu et al. 2021). However, morphological differences between A1 and A2 were not recognized based on leaf shape and size, trunk appearance, and flowers. Thus, the conflict between nuclear and chloroplast barcodes may be the consequence of cryptic evolutionary units and/or historical chloroplast introgression. Nevertheless, incomplete lineage sorting cannot be completely ruled out as an explanation for this phenomenon (Hollingsworth et al. 2011; Lu et al. 2016; Hu et al. 2019). In fact, the three well-delimited clusters (B, C, and D) can be directly distinguished from each other and from A based on the trunk appearance, while the other two morphological taxonomic scenarios failed in doing this (Fig. 1). However, none of these three scenarios could recognize the two chloroplast clusters (A1 and A2) within A. Hence, the third taxonomic scenario based on the trunk appearance (Fig. 1c) is better than the other two (Fig. 1a, b).

### ﻿Discrimination power and taxonomic implications

Usually, hybrid introgression and incomplete lineage sorting can induce a low discrimination power for DNA barcodes (Su et al. 2015; Lu et al. 2016, 2021). Nuclear ITS barcodes have shown strong discrimination power in many empirical studies (Pang et al. 2011; Wang et al. 2011; Su et al. 2015; Lu et al. 2016). In this study, both the nuclear ITS barcodes and the four chloroplast fragments combined showed high intraspecific discrimination power, although historical introgression between strains A and D might have occurred (Figs 3, 4, 7). However, a large discrepancy in the discrimination power of different chloroplast fragments was observed (Fig. 3b). For example, only *rbc*L could distinguish all chloroplast clusters, while the other three barcodes could only distinguish one from the remaining chloroplast clusters. Unsurprisingly, using DNA fragments with low discrimination power (e.g., *mat*K, *ycf*6-*trn*C, and *pet*A-*psb*J) to delimit clusters within this species did not yield accurate identification.

In consideration of the large differentiations based on both trunk appearance and molecular markers, whether the clusters in Fig. 1c and Fig. 7a should be treated as separate species or subspecies of Liang-Mian-Zhen remains to be evaluated with more substantial evidence based on genomic data, phenotypic traits, and crossing experiments. Regardless, ITS sequences and *rbc*L are proposed as barcodes for rapid and accurate identification of the different Liang-Mian-Zhen lineages in China.

### ﻿Molecular identification

Discrepancies in the safety and quality of medicinal materials from different species (or genetic clusters) have been demonstrated in many empirical studies (Bansal et al. 2018; Xin et al. 2018). In the present study, folk Liang-Mian-Zhen samples were shown to comprise several nuclear or chloroplast clusters with regional differences (Figs 3, 4, 7, Tables 1, 3), whereas cultivated Liang-Mian-Zhen material from a state-owned holding pharmaceutical listed company belong to just one of the clusters, suggesting the need to scientifically evaluate the safety and quality for different Liang-Mian-Zhen clusters or lineages. This study is the first to successfully reconcile nuclear molecular evidence (chloroplast conflict exists merely within cluster A) with the morphological taxonomy at the population level and to present accurate resource classification and molecular identification of Liang-Mian-Zhen in China. In summary, there are three major reasons for our accurate molecular identification within this species. First, barcodes with a high level of discrimination power were used to conduct taxonomic delimitation, and to carry out molecular identification. Second, extensive sampling helped to account for overlooked morphological variations and promoted the accuracy of taxonomic delimitation (Linck et al. 2019; Lu et al. 2021). Third, and most importantly, morphological classification or initial identification based on trunk appearance was further supported by integrating evidence from DNA barcodes and geographical distribution. In summary, accurate taxonomic delimitation is the basis for accurate and rapid molecular identification of closely related medicinal plants.

## ﻿Availability of data

All the data generated in this study are deposited in GenBank under the accession numbers MZ773282–MZ773393 and MZ769312–MZ769362.
